# Comparable performance of 3D and 2D anterior segment optical coherence tomography in predicting intraocular pressure reduction following cataract surgery

**DOI:** 10.1371/journal.pone.0345582

**Published:** 2026-03-25

**Authors:** Sunee Chansangpetch, Phichayut Phinyo, Jayanton Patumanond, Janejit Choovuthayakorn, Shan C Lin

**Affiliations:** 1 Center of Excellence in Glaucoma, Faculty of Medicine, Chulalongkorn University and King Chulalongkorn Memorial Hospital, Thai Red Cross Society, Bangkok, Thailand; 2 Department of Biomedical Informatics and Clinical Epidemiology (BioCE), Faculty of Medicine, Chiang Mai University, Chiang Mai, Thailand; 3 Center for Clinical Epidemiology and Clinical Statistics, Faculty of Medicine, Chiang Mai University, Chiang Mai, Thailand; 4 Department of Ophthalmology, Faculty of Medicine, Chiang Mai University, Thailand; 5 Glaucoma Center of San Francisco, San Francisco, California, United States of America; Sanmenxia Central Hospital, Henan University of Science and Technilogy, CHINA

## Abstract

**Purpose:**

To develop predictive models using three-dimensional (3D) and conventional two-dimensional (2D) anterior-segment optical coherence tomography (AS-OCT) parameters for intraocular pressure (IOP) reduction following phacoemulsification in glaucoma and non-glaucoma cohorts.

**Methods:**

This prospective study included patients with and without glaucoma who underwent phacoemulsification. Preoperative predictors of 1-month IOP reduction, including clinical, ocular biometry, and AS-OCT (CASIA2) parameters, were analyzed. 3D AS-OCT measurements were assessed in two approaches: (1) averaging 360-degree values and (2) estimating circumferential areas or volumes. 2D variables were obtained from horizontal cross-sectional images. Model selection with least absolute shrinkage and selection operator (LASSO) for 2D and 3D variables was performed separately. R-squared (R²) used to represent model accuracy. The performance was validated by bootstrap resampling.

**Results:**

A total of 130 eyes (64 glaucoma, 66 non-glaucoma) from 103 patients were included. The average IOP change was −1.20 ± 3.29 mmHg. Preoperative IOP (preIOP) was the strongest single predictor across all models. In the entire cohort, the final models showed moderate predictability (R² 38% for 3D and 36% for 2D; optimism-corrected R² 31% for both). In glaucoma eyes, models incorporating AS-OCT angle status and anterior chamber width achieved R² values of 36% (3D) and 38% (2D), with optimism-corrected R² of 27% (3D) and 28% (2D). In non-glaucoma eyes, both 3D and 2D models showed higher predictability (R² 53%, optimism-corrected R² 45%), with final predictors incorporating AS-OCT anterior chamber area and iris thickness. Both 3D and 2D models significantly outperformed the reference preIOP models across all cohorts (all p > 0.05).

**Conclusions:**

AS-OCT parameters improved the predictability of IOP reduction after phacoemulsification, with notably better performance in non-glaucoma eyes. 2D and 3D models showed comparable predictive ability.

## Introduction

Many studies have demonstrated that cataract extraction can successfully lower intraocular pressure (IOP). This effect has been established in patients with both narrow and open angles, with and without glaucoma [1–4]. The mechanism of IOP reduction involves widening of the anterior chamber angle following lens removal, particularly in eyes with narrow angles [5]. Changes in anterior segment features from lens removal can also lead to decreased IOP in patients with open angles [5,6].

Nevertheless, the IOP response following cataract surgery can be varied among individuals. Although some studies have shown no significant decrease in IOP [7,8], others have observed small but significant effects [9,10], and some have even found substantial reductions in IOP after surgery [4,11]. Accurately predicting the IOP response could assist clinicians in management planning for glaucoma or glaucoma suspect patients with concurrent cataracts. It can guide decisions on whether to perform cataract surgery alone or in combination with other glaucoma procedures in patients requiring IOP reduction, and help the clinician to assess the potential for reducing antiglaucoma medications after surgery.

Prior studies have found several characteristics that are associated with a decrease in IOP following cataract surgery. These factors include preoperative IOP level, presence of glaucoma, and angle status [12,13]. In addition, parameters representing anterior segment morphology have also been identified to be associated with degree of IOP changes. For example, a shallower preoperative anterior chamber depth (ACD) and a higher lens vault (LV) are associated with a more pronounced IOP reduction postoperatively [14,15]. Anterior segment optical coherence tomography (AS-OCT) imaging offers detailed assessment of anterior segment structures, including the angle and peripheral iris, and provides comprehensive morphometric information. These angle parameters have been shown to be associated with postoperative IOP variations [6,16,17]. However, most of these studies rely on two-dimensional (2D) cross-sectional AS-OCT images of horizontal scans (0º - 180º), capturing information only from the nasal and temporal angles.

CASIA2 (Tomey Corporation, Nagoya, Japan) is an AS-OCT device using swept-source technology. With an improvement in scan speed (50,000 A-scans per second) and axial resolution (10 µm), the device enables the acquisition of a more comprehensive image of the anterior chamber by capturing up to 128 frames circumferentially and interpolating the data between them, giving three-dimensional (3D) data. Consequently, the device can evaluate and quantify 3D parameters as well as 2D parameters across multiple meridians of the anterior chamber [18–20].

Since 3D scans provide detailed anatomical information, metrics derived from this technique may offer a more accurate prediction of IOP changes following cataract surgery than conventional 2D parameter measurements, potentially enhancing the predictability of postoperative IOP outcomes. This study aims to develop and validate a predictive model for IOP reduction after cataract surgery using metrics obtained from AS-OCT scans and to assess whether models developed from 3D measurements improved predictability compared to models developed from 2D measurements.

## Materials and methods

This prospective observational study was undertaken at the King Chulalongkorn Memorial Hospital, Bangkok, between 20 November 2022–30 September 2024. The study followed the Declaration of Helsinki and received approval from the Institutional Review Board of Chulalongkorn University (IRB 0698/65). All subjects provided written informed consent.

The study enrolled cataract patients, with or without primary glaucoma, aged 18 years or older who were scheduled for phacoemulsification with intraocular lens (IOL) implantation due to presence of visually significant cataract. Subjects with one or more of the following conditions were excluded: (1) history and/or evidence of ocular trauma including presence of lens subluxation, (2) previous glaucoma surgery, (3) presence of peripheral anterior synechiae in 6 clock hours or more, (4) secondary glaucoma including a history of steroid-induced ocular hypertension, (5) active intraocular inflammation within the past 3 months, (3) history of laser iridoplasty or laser trabeculoplasty, (6) presence of eyelid, corneal, or conjunctival abnormalities precluding adequate assessment of the anterior chamber by AS-OCT or accurate IOP measurement, or (7) inability to obtain qualified AS-OCT images.

Each participant received a comprehensive assessment at preoperative visits, which encompassed slit lamp examination, Goldmann applanation tonometry, gonioscopy, fundus examination, IOL calculation, and AS-OCT imaging. The demographic data and examination results were recorded. For biometry data, axial length (AL), anterior chamber depth (ACD), lens thickness (LT), and central corneal thickness (CCT) were obtained from optical biometry (IOLMaster 700; Carl Zeiss Meditec, Jena, Germany).

Gonioscopy was performed using a Sussman 4-mirror goniolens (Ocular Instruments Inc., WA, USA) under standardized dark-room conditions with ambient illumination <1 lux and a 1-mm narrow slit-lamp beam. A single glaucoma specialist (S.C.) conducted and recorded all examinations. Eyes were classified as having angle closure or an open angle based on the Asia-Pacific Glaucoma Guidelines [21]. Angle closure was defined as non-visualization of the posterior trabecular meshwork for ≥180° (≥2 quadrants), whereas eyes not meeting this criterion were categorized as open angle. Peripheral anterior synechiae (PAS) were documented by the number of clock hours involved. Primary angle-closure suspect (PACS) was defined as ≥180°of iridotrabecular contact without PAS, elevated IOP (>21 mmHg), or glaucomatous optic neuropathy. Primary angle closure (PAC) was diagnosed when PACS was accompanied by PAS and/or elevated IOP. Primary angle-closure glaucoma (PACG) was defined as PAC with evidence of glaucomatous optic neuropathy [22]. Diagnosis of glaucoma was based on the International Society of Geographic and Epidemiologic Ophthalmology (ISGEO) criteria [22]. Eyes classified as glaucoma suspects under ISGEO criteria were considered non-glaucoma except those suspected under the elevated IOP (ocular hypertension) or the PAC category, which were included in the glaucoma group for analysis.

### Anterior segment image acquisition

At the preoperative visit, all participants underwent anterior segment imaging using the CASIA2 (Tomey Corporation, Nagoya, Japan) by a single experienced operator. For image capture, either the ‘Global AC Analysis’ or ‘Lens Global Analysis’ mode was used. Both modes comprise 128 consecutive radial scans (800 A-scans per line) covering 360 degrees of the entire anterior segment. An assistant gently held the participants’ eyelids to avoid applying pressure on the eye. Scans were performed under dim-light conditions (<1 lux). Each scan was assessed for image quality immediately by the operator. Scans were repeated in cases of eyelid artifacts, motion artifacts, or insufficient visibility of the anterior chamber. If a scan meeting the quality criteria could not be obtained after three attempts, the patient was excluded from the study.

### Anterior segment image analysis

Interfaces on B-scan images were automatically outlined by the built-in ‘STAR 360’ software (version 3A.4). The software calculates the parameters by utilizing 16 evenly-spaced section images (32 angles) after manual confirmation or adjustment of the scleral spur (SS) locations. A glaucoma specialist (S.C.) conducted a thorough review of all images, where subject identifications were masked, to evaluate the SS and angle recess (AR) positions, as well as all boundaries. If the image was determined to have an incorrect SS or AR location, a manual adjustment was made.

The software automatically calculated anterior segment parameters for each image. The parameters comprised lens vault (LV), iris area (Iarea), iris curvature (Icurv), and iris thickness (IT), as well as parameters representing anterior segment dimensions, including anterior chamber width (ACW) and anterior chamber area (ACarea), and angle parameters. Iris thickness was assessed at 750 µm and 2,000 µm from the scleral spur, resulting in 2 iris parameters (IT750 and IT2000). Angle parameters included angle opening distance (AOD), angle recess area (ARA), and trabecular iris space area (TISA). All angle parameters were assessed at 250, 500, and 750 µm from the scleral spur, resulting in 9 angle parameters. The detailed definition of each parameter was described in previous literature [23].

Except for LV which was measured in an axial direction, each of the AS-OCT derived parameters was assessed using two spatial approaches: 2D and 3D.

- 2D – The values obtained from the horizontal image (*-hoz*) at the 0º - 180º meridian were used for the 2D models. For angle and iris parameters that have two values in a single image, the nasal and temporal values were averaged.
- 3D – This set of parameters incorporated 360-degree information from all image meridians. Two types of 3D parameters were automatically computed and exported for the analysis: (1) average parameter (*-avg*) – the averaged value from 360-degree values and (2) estimate parameter (*-est*) – the estimation of circumferential area (e.g., surface area of circumferential angle inlet for AOD, circumferential cross-sectional area for IT); or circumferential volume (for ACarea, Iarea, ARA, TISA).

**Fig 1:**
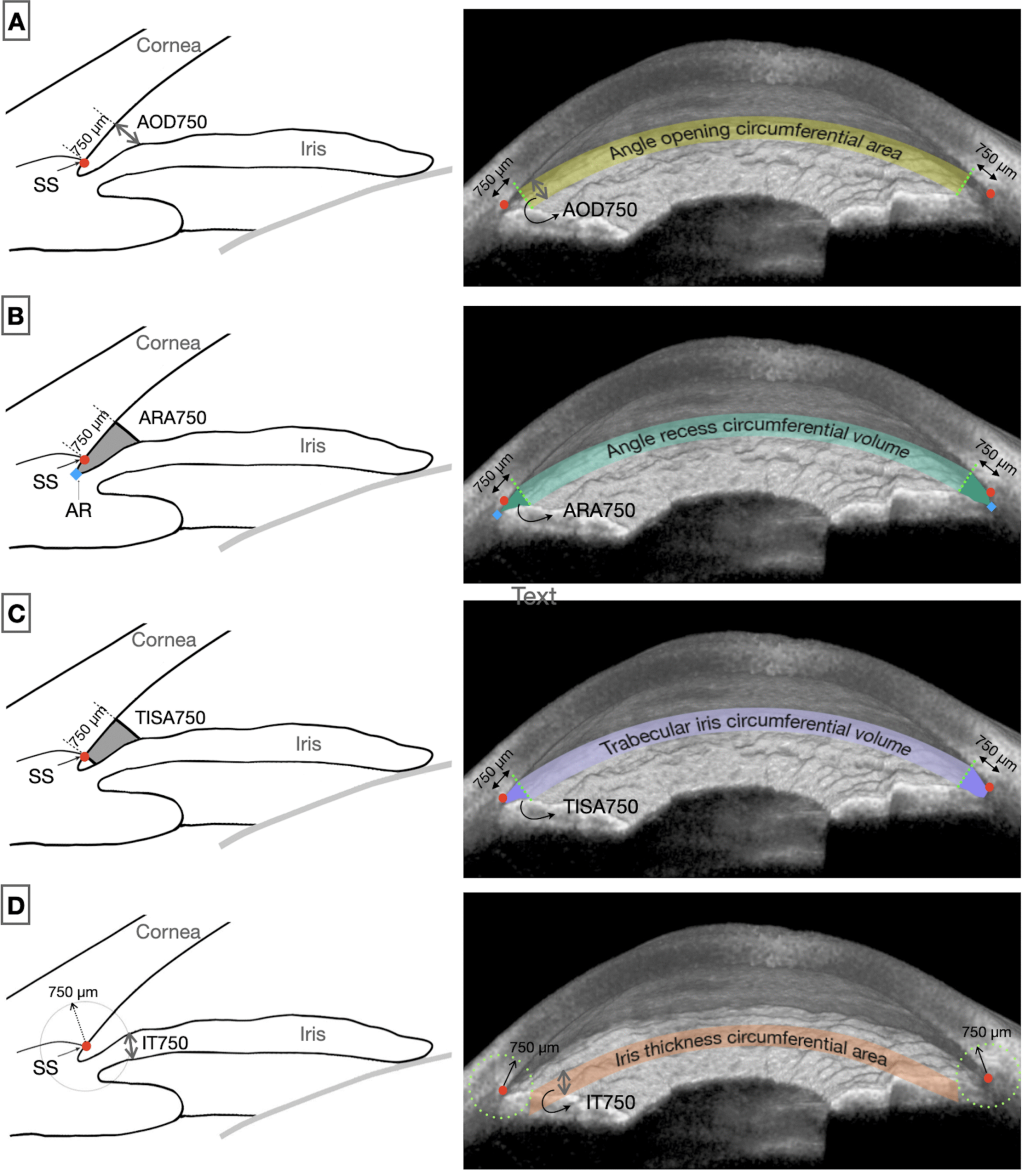
Illustration of 2D and 3D angle and iris parameters at 750 µm from SS. Schematic 2D parameters of **(A)** AOD, **(B)** ARA, **(C)** TISA, **(D)** IT are shown in the left panel. The 3D average parameters (-avg) are the average of 360-degree 2D measurements. The corresponding 3D estimate parameters (-est) on the 3D display of AS-OCT images are shown in the right panel, including **(A)** AOD750-est or angle opening circumferential area, **(B)** ARA750-est or AR circumferential volume, **(C)** TISA750-est or trabecular iris circumferential volume, and **(D)** IT750-est or iris thickness circumferential area; SS, scleral spur (red circle); AR angle recess (blue diamond); AS-OCT, anterior segment–optical coherence tomography; 2D, two-dimensional; 3D, three-dimensional; AOD, angle opening distance; ARA, angle recess area; TISA, trabecular iris space area; IT, iris thickness.

The ‘average’ type was generated for all AS-OCT morphometrics parameters. The ‘estimate’ type was generated for ACarea (equivalent to anterior chamber volume), Iarea (equivalent to iris volume), IT, and all angle parameters. The circumferential angle inlet of AOD can be referred to as angle opening circumferential area. The circumferential angle volumes for ARA and TISA are respectively known as angle recess circumferential volume and trabecular-iris circumferential volume [18,20]. The detailed description of 3D parameters has been published elsewhere [19]. Analogous to the angle opening circumferential area derived from AOD, the circumferential area corresponding to iris thickness can be described as the iris thickness circumferential area. Fig 1 shows examples of 2D and 3D AS-OCT parameters.

### Surgery and follow up

All patients underwent standard phacoemulsification with posterior chamber IOL implantation under topical anesthesia through a temporal clear corneal incision, performed by the same surgeon (S.C). The IOL models used were SA60WF (Alcon, Fort Worth, TX, USA) for non-toric cases and AT TORBI 709M/MP (Carl Zeiss Meditec, Jena, Germary) for toric cases. A fixed-combination eyedrop containing tobramycin and dexamethasone was used four times a day for two weeks after surgery, and then tapered off. Follow-up appointments were scheduled one day, one week, and one month after surgery. To avoid the confounding effect of adjusting glaucoma medication on the IOP outcome, patients who used antiglaucoma drugs were given the same regimen for one month after surgery, unless the IOP decreased below 6 mmHg or increased from baseline to above 30 mmHg. Postoperative IOP was obtained at the 1-month visit. Complicated cataract surgery was excluded from the analysis.

### Model outcome

The outcome of the study was IOP reduction after phacoemulsification at 1 month of follow-up. IOP change was defined as postoperative IOP at 1 month minus preoperative IOP (minus values indicate IOP reduction).

### Variable preparation

The relationship between IOP change and all possible predictors were initially assessed using univariable linear regression with a cluster-robust variance estimator (CRVE). This method adjusts the standard errors to account for the correlation between eyes from the same individual, thereby preventing underestimated standard errors and inflated statistical significance [24]. The linearity assumption between IOP change and each continuous predictor was assessed using locally weighted scatterplot smoothing (LOWESS) curves. Due to visibly non-linear patterns, lens thickness and all angle parameters were dichotomized to improve model stability and simplify interpretation. All 2D and 3D angle variables were categorized to represent closed and open angles using a previously published diagnostic cutoff for angle closure [19]. Values greater than the cutoff indicated an open angle. Additionally, lens thickness was categorized, with values greater than 5 mm classified as a thick lens.

**Fig 2:**
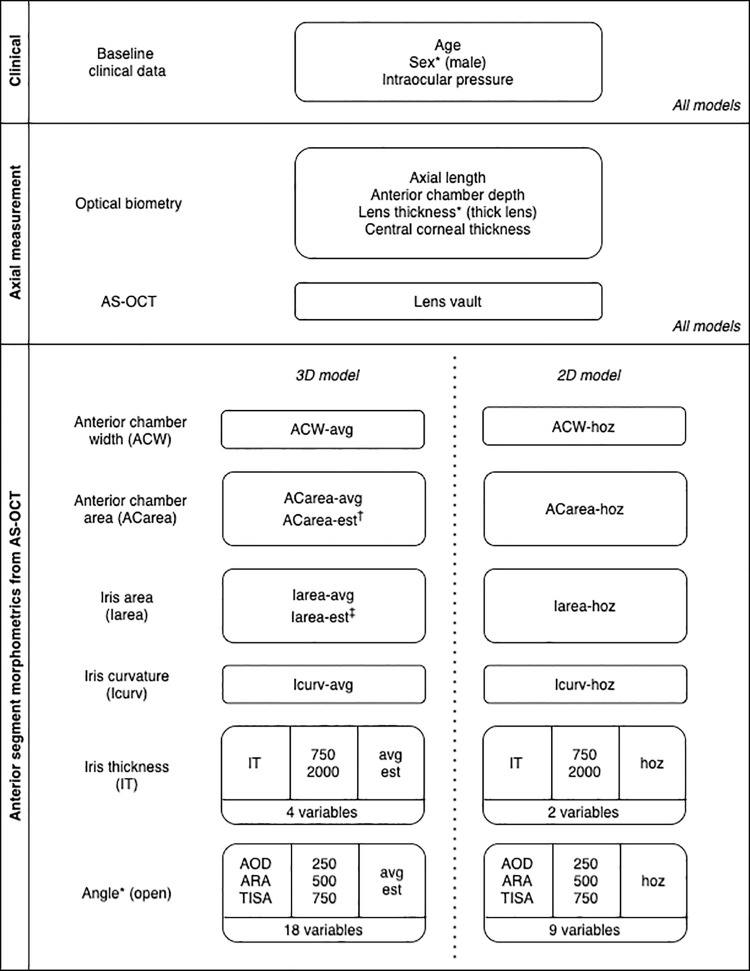
Diagram summarizing all potential predictors categorized into clinical data, axial measurement and anterior segment morphometrics. AS-OCT = anterior segment optical coherence tomography; AOD = angle opening distance; ARA = angle recess area; TISA = trabecular iris space area; -avg = average of 360-degree angle values; -est = estimation of circumferential area (for IT and AOD) or circumferential volume (for ACarea, Iarea, ARA, and TISA); -hoz = horizontal meridian (average of nasal and temporal sides); * binary factors – the value in parentheses indicates the represented category; † equivalent to anterior chamber volume; ‡ equivalent to iris volume.

The potential predictors comprised 23 variables in the 2D analysis and 36 variables in the 3D analysis. These variables were categorized into three main groups: (1) clinical data (3 variables), (2) axial measurement (5 variables), and (3) anterior segment morphometric, consisting of 15 variables in 2D and 28 variables in 3D. Variables from the ‘clinical data’ and ‘axial measurement’ categories were incorporated into both the 2D and 3D models. Variables within the ‘anterior segment morphometric’ category were included in model development separately, based on their dimensional type. Fig 2 summarizes a list of variables grouped by category.

### Model development

The 2D and 3D models were separately developed employing a linear regression with least absolute selection and shrinkage operator (LASSO) penalization for variable selection, using 10-fold cross-validation. From each fold, the most frequently selected predictors based on the minimum lambda were identified across the regularization path. A frequency threshold of 0.6 (i.e., selection in more than 6 out of 10 folds) was used to retain stable predictors for further modeling [25]. In cases where multiple predictors from the same anatomical category of anterior segment morphometrics were selected (e.g., angle category or iris thickness category), only the more frequently selected variable was retained. The models were further refined by stepwise reduction using removal thresholds of 0.3. Borderline predictors that did not meet the initial stability selection threshold but demonstrated an improvement in model fit were subsequently included in the final model refinement. This process resulted in the final 2D and 3D models. The cross-validation incorporated subject-level splitting to mitigate within-subject correlation. Model development was then performed in the subgroups of eyes with and without glaucoma.

### Model performance

The final models, developed separately for 2D and 3D parameters, were fitted using the full dataset. Regression models were built with a CRVE. Model performance was assessed using the coefficient of determination (R^2^), adjusted R^2^, and root mean squared error (RMSE). Internal validation was conducted using 500 bootstrap resamples to estimate optimism-corrected performance metrics.

The performance was then compared across models. Given that preoperative IOP (preIOP) was the strongest single predictor of postoperative IOP, as supported by previous literature [12,26], a preIOP model was built as a comparative reference to evaluate the added value of our predictive models. Model performance was tested against the baseline IOP model using the likelihood-ratio test. Two additional predictive formulas, developed in our dataset using the same approach based on preIOP/ACD and preIOP × ACD as previously proposed in the literature [14,27], were included for reference. To evaluate whether the inclusion of AS-OCT parameters enhanced predictive performance to the clinical variables, we also built clinical-only models as another reference set. These models included only the predictors retained in the final model that originated from the clinical category. The same assessment of performance was applied for each glaucoma and non-glaucoma subgroup. For each subgroup, the clinical-only model consisted of the clinical-category predictors retained in that subgroup’s final model.

### Statistical analysis

The sample size was estimated using Riley’s method for developing a prediction model with a continuous outcome [28]. Parameters were based on a prior study: expected R-squared 0.56, intercept 2.55, and standard deviation 1.78 [14]. The number of candidate predictors was set at 14, representing 14 distinct types of features. A minimum of 128 eyes was required to ensure minimal overfitting (expected shrinkage by 10% or less) and optimism (adjusted R^2^ optimism less than 0.05).

Baseline characteristics were summarized using descriptive statistics. Comparisons between the glaucoma and non-glaucoma subgroups were conducted using the independent t-test or Mann–Whitney U test for continuous variables, and the chi-square test or Fisher’s exact test for categorical variables. Preoperative and 1-month postoperative outcomes were compared using paired t-tests. Data management and analysis were performed using Stata/MP 16.0 (StataCorp LLC, College Station, TX, USA) and R 4.4.0 (R Foundation for Statistical Computing, Vienna, Austria).

## Results

The study enrolled 147 eyes from 120 participants. Three eyes were excluded due to intraoperative zonule weakness, and fourteen eyes were excluded due to poor visibility of the scleral spurs. The final analysis included 130 eyes from 103 participants. Ninety-seven eyes received a monofocal non-toric IOL, and 33 eyes received a toric IOL. Among these, 34 eyes (26.2%) were angle closure, and 64 eyes (49.2%) had a diagnosis of glaucoma. PAS was present in 10 eyes, all of which were PACG, with a median extent of 3.5 clock hours (interquartile range 2.25 to 5.25). Eyes with glaucoma had more angle closure proportion, shorter AL, smaller ACD, higher LV, smaller anterior chamber area, and narrower angle width than eyes without glaucoma. For eyes with glaucoma, the average number of preoperative glaucoma medications was 1.5 ± 1.4. Clinical, ocular biometry, and selected AS-OCT parameters of all subjects, glaucoma and non-glaucoma groups are shown in Table 1. Diagnosis subtypes are shown in Supplementary S1 Fig. The complete values of all variables are detailed in Supplementary S1, S2, and S3 Tables.

**Table 1:** Baseline clinical data, ocular biometry, and anterior segment dimensions from AS-OCT.

	All	Glaucoma	Non-glaucoma	P value
Eye (patient)	130 (103)	64 (51)	66 (52)	
**Clinical data**				
Age (year)	71.0 ± 8.7	70.6 ± 8.2	70.5 ± 9.7	0.98
Sex				0.20
Male	42 (40.8)	24 (47.1)	18 (34.6)	
Female	61 (59.2)	27 (52.9)	34 (65.4)	
Laterality				0.014
Right	71 (54.6)	28 (43.8)	43 (65.2)	
Left	59 (45.4)	36 (56.2)	23 (34.8)	
Angle status				0.012
Open angle	96 (73.8)	41 (64.1)	55 (83.3)	
Closed angle	34 (26.2)	23 (35.9)	11 (16.7)	
Preoperative IOP (mmHg)	14.2 ± 3.7	14.4 ± 4.4	14.0 ± 2.8	0.46
**Axial measurement**				
Axial length (mm)	23.74 ± 1.26	23.39 ± 0.95	24.08 ± 1.43	0.002
Anterior chamber depth (mm)	2.98 ± 0.60	2.82 ± 0.65	3.14 ± 0.51	0.002
Lens thickness (mm)	4.77 ± 0.51	4.84 ± 0.48	4.71 ± 0.52	0.14
Lens vault (mm)	0.45 ± 0.49	0.62 ± 0.51	0.28 ± 0.40	<0.001
Central corneal thickness (µm)	523.90 ± 32.12	521.06 ± 31.90	526.65 ± 32.33	0.32
**3D anterior segment morphometrics**				
ACW – *avg* (mm)	11.37 ± 0.41	11.37 ± 0.40	11.37 ± 0.43	0.96
ACarea – *avg* (mm^2^)	18.09 ± 4.50	16.76 ± 4.53	19.38 ± 4.10	<0.001
ACarea– *est* (AC volume) (mm^3^)	117.64 ± 34.30	108.87 ± 34.31	126.14 ± 32.33	0.004
Iarea – *avg* (mm^2^)	1.518 ± 0.217	1.501 ± 0.205	1.534 ± 0.228	0.38
Iarea – *est* (iris volume) (mm^3^)	35.19 ± 4.31	35.09 ± 3.93	35.29 ± 4.69	0.79
IC – *avg* (mm)	0.175 ± 0.095	0.165 ± 0.082	0.184 ± 0.106	0.26
IT750 – *avg* (mm)	0.373 ± 0.059	0.371 ± 0.052	0.375 ± 0.066	0.71
IT750 – *est* (mm^2^)	11.17 ± 2.21	11.01 ± 2.16	11.33 ± 2.27	0.41
AOD750 – *avg* (mm)	0.434 ± 0.262	0.372 ± 0.217	0.493 ± 0.288	0.007
AOD750 – *est* (mm^2^)	13.21 ± 7.81	11.40 ± 6.63	14.96 ± 8.48	0.009
**2D anterior segment morphometrics**				
ACW – *hoz* (mm)	11.30 ± 0.43	11.31 ± 0.41	11.30 ± 0.44	0.85
ACarea – *hoz* (mm^2^)	18.29 ± 4.47	16.95 ± 4.51	19.58 ± 4.07	<0.001
Iarea – *hoz* (mm^2^)	1.447 ± 0.217	1.435 ± 0.205	1.459 ± 0.229	0.53
IC – *hoz* (mm)	0.179 ± 0.101	0.168 ± 0.090	0.189 ± 0.110	0.25
IT750 – *hoz* (mm)	0.369 ± 0.069	0.367 ± 0.063	0.371 ± 0.075	0.71
AOD750 – *hoz* (mm)	0.471 ± 0.290	0.403 ± 0.234	0.537 ± 0.323	0.008

Data shown in N (%) and mean ± standard deviation; IT750 and AOD750 are shown to represent iris thickness and angle parameters, respectively; IOP = intraocular pressure; 3D = three-dimensional; 2D = two-dimensional; ACW = anterior chamber width; ACarea = anterior chamber area; Iarea = iris area; IC = iris curvature; IT = iris thickness; AOD = angle opening distance; -avg = 3D average parameter; -est = 3D estimate parameter

After cataract surgery, the mean (standard deviation, SD) IOP significantly decreased from 14.2 (3.7) mmHg at baseline to 12.9 (3.5) mmHg (p = 0.001) at one month in the entire cohort. The glaucoma group showed a significant reduction from 14.4 (4.4) mmHg to 13.1 (4.3) mmHg (p = 0.002), and the non-glaucoma group from 14.0 (2.8) mmHg to 12.9 (2.5) mmHg (p = 0.009). None of the eyes in the glaucoma group experienced an IOP below 6 mmHg or an increase above 30 mmHg during the study; therefore, all patients remained on the same glaucoma medication regimen. Visual acuity also significantly improved for the entire cohort, from 0.70 (0.57) logMAR preoperatively to 0.19 (0.23) logMAR postoperatively (p < 0.001). Similarly, visual acuity improved from 0.65 (0.53) to 0.22 (0.22) logMAR in the glaucoma group (p < 0.001), and from 0.76 (0.60) to 0.15 (0.24) logMAR in the non-glaucoma group (p < 0.001).

For the entire cohort, the mean (SD) change in IOP at 1-month post-operation was −1.21 (3.29) mmHg from baseline, with a maximum IOP reduction of −13 mmHg. Additionally, 42 (32.3%) eyes experienced an IOP decrease of more than −3 mmHg. The mean (SD) changes were −1.32 (3.30) mmHg in the glaucoma group and −1.09 (3.29) mmHg in the non-glaucoma group.

### Univariable analyses

The analysis in the entire cohort showed that age (coefficient [β] −0.08, 95% CI −0.13 to −0.02) and preIOP (β −0.44, 95% CI −0.66 to −0.23) were significantly associated with 1-month IOP change (p < 0.05). In addition, Iarea-avg, ACW-hoz, and open-angle status as determined by AOD750-avg were marginally associated with 1-month IOP change with a p-value of less than 0.2 (Table 2 and Supplementary S1 Table).

**Table 2:** Univariable analysis of selected predictors for 1-month intraocular pressure change after phacoemulsification.

	All			Glaucoma			Non-glaucoma		
	Coefficient	P-value	95% CI	Coefficient	P-value	95% CI	Coefficient	P-value	95% CI
*Clinical data*									
Age	−0.075	**0.012**	−0.132 to −0.017	−0.076	0.129	−0.175 to 0.023	−0.075	0.044	−0.147 to -0.002
Sex (male)	−0.574	0.346	−1.777 to 0.628	−0.714	0.384	−2.341 to 0.914	−0.372	0.707	−2.336 to 1.593
preIOP	−0.441	**<0.001**	−0.655 to −0.227	−0.297	**<0.001**	−0.452 to −0.141	−0.772	**<0.001**	−1.009 to −0.535
*Axial measurement*									
Axial length	0.010	0.971	−0.556 to 0.577	−0.698	0.037	−1.353 to -0.044	0.270	0.486	−0.500 to 1.041
CCT	0.000	0.965	−0.018 to 0.017	0.000	0.990	−0.027 to 0.027	−0.001	0.920	−0.026 to 0.023
*3D anterior segment morphometrics*									
ACW*-avg*	0.831	0.285	−0.701 to 2.362	1.238	0.229	−0.801 to 3.277	0.481	0.676	−1.805 to 2.767
ACarea*-avg*	0.018	0.854	−0.174 to 0.210	−0.106	0.081	−0.292 to 0.261	0.149	0.408	−0.209 to 0.507
AOD750*-avg*	−1.078	0.150	−2.551 to 0.396	−2.005	**0.042**	−3.932 to −0.078	0.109	0.919	−2.022 to 2.240
IT750*-avg*	5.076	0.257	−3.745 to 13.897	−0.379	0.964	−17.102 to 16.344	8.225	0.124	−2.308 to 18.757
Iarea*-avg*	−2.011	0.184	−4.992 to 0.970	−1.947	0.381	−6.357 to 2.463	−2.160	0.290	−6.209 to 1.889
*2D anterior segment morphometrics*									
ACW*-hoz*	1.082	0.176	−0.490 to 2.654	1.663	0.164	−0.695 to 4.020	0.602	0.583	−1.579 to 2.784
ACarea*-hoz*	0.019	0.847	−0.172 to 0.209	−0.106	0.257	−0.291 to 0.079	0.152	0.391	−0.199 to 0.502
AOD750*-hoz*	−0.832	0.254	−2.269 to 0.606	−1.802	**0.064**	−3.714 to 0.111	0.398	0.698	−1.644 to 2.440
IT750*-hoz*	2.272	0.585	−5.929 to 10.472	−0.796	0.908	−14.515 to 12.923	4.267	0.426	−6.378 to 14.912

Only predictors retained in the final models are shown. IOP = intraocular pressure; 3D = three-dimensional; 2D = two-dimensional; CCT = central corneal thickness; ACW = anterior chamber width; ACarea = anterior chamber area; Iarea = iris area; IT = iris thickness; AOD = angle opening distance; -avg = 3D average parameter; -hoz = 2D horizontal parameter

In the glaucoma subgroup, preIOP (β −0.30, 95% CI −0.45 to −0.14) and open angle status determined by AOD750-avg (β −2.01, 95% CI −3.93 to −0.08) and AL (β −0.70, 95% CI −1.35 to −0.04) were significantly associated with 1-month IOP change (p < 0.05). Marginal associations (p < 0.2) were found for age, LV, ACW-hoz, Icurv (-avg and -hoz), and open-angle status determined by AOD750 (3D-est and 2D-hoz), ARA750-hoz, and TISA750-hoz (Table 2 and Supplementary S2 Table).

In the non-glaucoma subgroup, preIOP (β −0.77, 95% CI −1.01 to −0.54) and thick lens (β −1.87, 95% CI −3.50 to −0.24) were significantly associated with 1-month IOP change (p < 0.05). Marginal associations (p < 0.2) were observed for age and IT750 (both 3D -avg and -est) (Table 2 and Supplementary S3 Table).

### Prediction models

All final model formulas are shown in Table 3. The full model details are shown in Supplementary S4 Table. The retained variables at each step are described below.

**Table 3:** Final model formulas for predicting 1-month intraocular pressure change after phacoemulsification.

Cohort	Model	Final model formulas
All subjects	3D	−6.61–0.48 *preIOP -* 0.07 *age –* 1.19 *sex (male)* – 1.35 *AOD750-avg* *(open)* +1.05 *ACW-avg* + 0.01 *CCT* + 4.87 *IT750-avg -* 1.74 *Iarea-avg*
	2D	−5.93–0.48 *preIOP -* 0.08 *age –* 1.18 *sex (male)* – 1.26 *AOD750-hoz* *(open)* +1.04 *ACW-hoz* + 0.01 *CCT*
Glaucoma	3D	−3.12–0.33 *preIOP* - 0.06 *age –* 0.76 *AL* – 1.89 *AOD750-avg* *(open)* +1.92 *ACW-avg* + 0.02 *CCT*
	2D	−7.32–0.34 *preIOP* - 0.05 *age* – 0.88 *AL* – 1.71 *AOD750-hoz* *(open)* +2.40 *ACW-hoz* + 0.02 *CCT*
Non-Glaucoma	3D	−3.86–0.82 *preIOP* + 0.16 *ACarea-avg + 5.88 IT750-avg* + 0.02 *CCT*
	2D	−4.52–0.83 *preIOP* + 0.19 *ACarea-hoz + 5.67 IT750-hoz* + 0.02 *CCT*

In the entire cohort, LASSO regression identified predictors for the 3D model: preIOP, age, AOD750-avg, sex, IT750-avg, CCT, Iarea-avg, TISA250-est, and ACW-avg. TISA250-est was excluded due to lower selection frequency than AOD750-avg, which was in the same angle category. Stepwise regression yielded a final eight-predictor model. For the 2D model, initial predictors were preIOP, age, AOD750-hoz, sex, ACW-hoz, CCT, and ARA500-hoz. ARA500-hoz was similarly removed, and stepwise regression did not further reduce the model, resulting in a final six-predictor model.

In the glaucoma subgroup, stability selection identified preIOP, age, AOD750-avg, LV, AL, and ACW-avg for the 3D model. Stepwise regression removed LV, but adding the borderline variable CCT improved adjusted R² and RMSE, yielding a final six-predictor model. The 2D model initially included preIOP, AOD750-hoz, ACW-hoz, age, LV, AL, sex, and CCT. Stepwise regression removed LV and sex, leaving a final six-predictor model.

In the non-glaucoma subgroup, initial predictors for the 3D model were preIOP, age, ACarea, IT750-avg, LT, and LV. Stepwise regression removed age, LT, and LV, and the addition of CCT improved performance, resulting in a final four-predictor model. The 2D model began with preIOP, age, ACarea-hoz, LT, LV, and IT750-hoz. Stepwise regression removed age, LV, and LT, and again, adding CCT improved model fit, leading to a final four-predictor model.

### Model performance and validation

Model performance metrics are summarized in Table 4. In the entire cohort, the 3D model showed the highest apparent R² (38.0%) and lowest RMSE (2.672), with optimism-corrected R² of 31.0% and RMSE of 2.792. Both the 3D and 2D models significantly outperformed the baseline IOP model (3D model p = 0.003, 2D model p = 0.002). The predictive models demonstrated notably better performance in the non-glaucoma subgroup compared to the glaucoma subgroup. In non-glaucoma eyes, the 2D model achieved the highest apparent R² (53.0%) and lowest RMSE (2.331), with optimism-corrected R² of 45.3% and RMSE of 2.506. In contrast, model performance dropped in the glaucoma subgroup, with the 2D model showing an apparent R² of 37.5% and optimism-corrected R² of 28.1%, and a higher RMSE of 2.884. Nevertheless, in both subgroups, the 3D (glaucoma p = 0.003, non-glaucoma p = 0.015) and 2D (glaucoma p = 0.002, non-glaucoma p = 0.011) models significantly outperformed the baseline IOP model, with comparable performance between the 3D and 2D models. In addition, both the 3D and 2D models significantly outperformed their corresponding clinical models in the entire cohort (3D model p = 0.007, 2D model p = 0.009) and in the glaucoma subgroup (3D model p = 0.004, 2D model p = 0.002). For non-glaucoma subgroup, the clinical model was identical to the baseline IOP model, which both the 3D and 2D models also outperformed.

**Table 4:** Comparison of model performance.

Cohort	Model	Apparent			Optimism-corrected		
R2	Adjusted R2	RMSE	R2	Adjusted R2	RMSE
All subjects	preIOP	0.243	0.237	2.871	0.235	0.229	2.929
	preIOP x ACD	0.172	0.165	3.003	0.177	0.170	3.042
	preIOP ÷ ACD	0.085	0.078	3.155	0.072	0.064	3.219
	Clinical model^a^	0.299	0.282	2.784	0.271	0.265	2.862
	3D	0.380	0.339	2.672	0.310	0.303	2.792
	2D	0.359	0.327	2.696	0.305	0.298	2.799
Glaucoma	preIOP	0.155	0.141	3.058	0.142	0.129	3.121
	preIOP x ACD	0.140	0.126	3.085	0.129	0.115	3.150
	preIOP ÷ ACD	0.034	0.018	3.270	0.013	0.002	3.341
	Clinical model^b^	0.184	0.157	3.029	0.151	0.137	3.111
	3D	0.360	0.293	2.775	0.265	0.249	2.913
	2D	0.375	0.309	2.744	0.281	0.266	2.884
Non-glaucoma	preIOP	0.444	0.435	2.477	0.445	0.436	2.529
	preIOP x ACD	0.225	0.213	2.923	0.219	0.206	2.995
	preIOP ÷ ACD	0.334	0.324	2.709	0.330	0.320	2.750
	Clinical model^c^	0.444	0.435	2.477	0.445	0.436	2.529
	3D	0.525	0.494	2.343	0.448	0.437	2.518
	2D	0.530	0.499	2.331	0.453	0.443	2.506

preIOP = pre-operative intraocular pressure; ACD = anterior chamber depth; 3D = three-dimensional model; 2D = two-dimensional model; R2 = coefficient of determination; RMSE root mean square error

^a^Clinical model for All subjects consists of age preIOP and sex.

^b^Clinical model for Glaucoma subgroup consists of age and preIOP.

^c^Clinical model for Non-glaucoma subgroup consists of preIOP.

We found no superiority in the performance of the models based on preIOP × ACD and preIOP/ACD compared with our models in the entire cohort or any subgroup. Because these two reference models were not nested within ours, likelihood ratio tests could not be performed.

**Fig 3:**
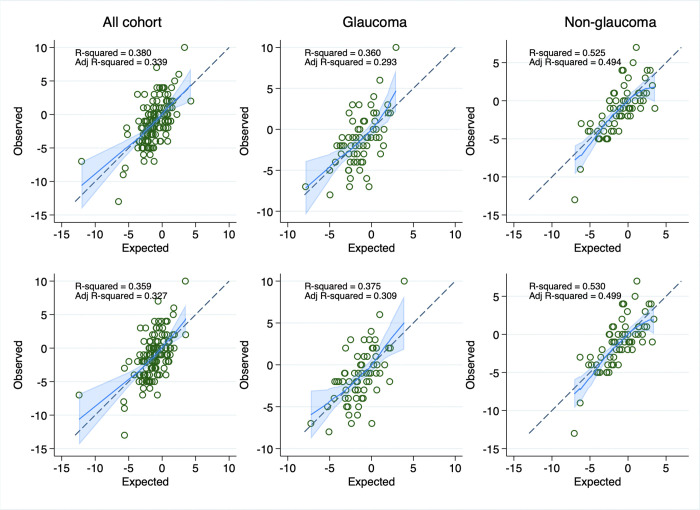
Calibration plots showing predicted versus observed 1-month postoperative IOP. The upper row represents 3D models and the bottom row 2D models. The blue lines show smoothed calibration curves, and the light blue shaded bands represent the 95% confidence intervals. The 45-degree dashed lines indicate perfect calibration.

Calibration plots showed good agreement between predicted and observed postoperative IOP for both 3D and 2D models. Predictions in the non-glaucoma subgroup were more closely aligned with observed values compared to the glaucoma subgroup. The calibration plots of 3D and 2D models are shown in Fig 3.

## Discussion

In this prospective study, we developed predictive models to estimate 1-month postoperative IOP change after phacoemulsification using baseline variables on clinical, biometry, and anterior segment morphometrics. We built models separately for novel 3D variables and horizontal 2D variables. The final models showed moderate predictive performance in overall cohorts with R² of 38% (optimism-corrected R² 31%) for the 3D model and 36% (optimism-corrected R² 31%) for the 2D model. The performance was substantially higher in the non-glaucoma subgroup with R² of 53% (optimism-correct R² 45%) for the 3D model and 53% (optimism-corrected R² 45%) for the 2D model. Our findings demonstrate that the 2D and 3D models performed comparably in predicting IOP reduction after phacoemulsification.

The advancement of imaging technology allows 3D measurements of anterior segment which give greater details about overall dimensions compared to the conventional 2D measurements. Previous studies showed that anterior chamber space is not evenly distributed and can vary significantly [29–31]. Using a single 2D image might be inadequate to represent the whole anterior chamber. We hypothesized that 3D parameters might add benefits in predicting IOP change. Clinical data, baseline biometry, and variables from AS-OCT that showed anterior chamber dimensions (i.e., anterior chamber depth, width, area, and volume) and AS-OCT variables that represents structures possibly relevant to IOP change including lens, iris, and angle factors were selected to form predictive formulas. The initial potential predictors started off with large sets of variables – 36 variables in 3D model and 23 variables in 2D models. To exclude a less relevant predictor, the LASSO regression was applied for variable selection. LASSO regression penalizes large coefficients and shrinks some coefficients to be zero, leading to a simpler model with fewer important features, and thus, reducing overfitting.

Despite using LASSO and stepwise regression, the initial modeling using the entire cohort retained a large number of variables, failing to yield a sufficiently parsimonious model with desired performance. This may be attributed to overlapping information among predictors and the absence of a small set of strongly predictive features. However, the model built on non-glaucoma subjects resulted in a small model with substantially increased performance. Supporting our findings, previous studies had also shown that presence of glaucoma influences predictability of IOP change. [12,32] This suggests that, aside from preIOP, the AS-OCT and biometric variables being studied may not sufficiently explain or predict IOP changes in glaucomatous eyes, in contrast to non-glaucomatous eyes.

Across all models and subgroups, high preIOP remained the most robust and consistent predictor of postoperative IOP reduction, aligning with previous reports in the literature [12,14,26,27,32]. Previous studies demonstrated that IOP in combination with ACD could predict IOP reduction after cataract surgery. Issa et al. built a model with a composite variable, IOP/ACD, as a predictor to estimate IOP reduction at 8 weeks in non-glaucomatous eyes [14]. The model demonstrated R^2^ of 73%. Liu et al. estimated the IOP change in primary angle closure eyes with another composite variable, IOP x ACD, and found that it can explain 49% of IOP variation at 1 year after surgery [27]. We built three reference models using these variables – IOP, IOP/ACD, and IOP x ACD – to compare the performances with our models. When fitted to our current dataset, lower R^2^ values compared to the original reports were expected. Despite the low R^2^, these reference models showed a similar trend to our models, with the highest predictability in the non-glaucoma group and the lowest in the glaucoma group. However, our models clearly improve predictability in the current dataset with increased R^2^ and lower RMSE compared to the three reference models, proving the added value of imaging and anterior segment morphometric parameters.

From univariable analysis and feature selection from the LASSO method, we found that different groups of variables contributing to IOP change differently in different cohorts. For glaucoma eyes, angle status—particularly determined by AOD750— and ACW demonstrated the predictive value among AS-OCT parameters in glaucoma eyes, whereas parameters representing iris and lens features were not selected in this group. In contrast, both ACW and all angle parameters were not selected in the predictive models of non-glaucoma cohorts. ACarea and IT750 were found to be the most predictive AS-OCT in non-glaucoma eyes. These differences underscore the need to consider distinct anatomical predictors when modeling IOP response in eyes with different glaucoma status.

The predictive ability of the models was lower in eyes with a glaucoma diagnosis compared to those without. This may reflect the influence of other factors, such as trabecular outflow function, not captured by anterior segment imaging. Interestingly, in our glaucoma cohort, the final model showed that open-angle status was associated with greater IOP reduction after cataract surgery compared to closed angle, after adjusting for other parameters. This finding may reflect chronic structural damage in angle-closure eyes, such as long-standing trabecular dysfunction, which may limit the functional improvement in aqueous outflow despite anatomical angle widening after cataract surgery. In contrast, eyes with open angles may retain more responsive outflow pathways, allowing for more effective IOP reduction following phacoemulsification. These observations highlight that while anatomical changes are important, the underlying functional integrity of the outflow system may also influence IOP response to cataract surgery. Future research to explore this issue is warranted.

According to the final models for non-glaucoma eyes, a small anterior chamber, as determined by ACarea, was associated with greater IOP reduction. Eyes with a thick peripheral iris, as indicated by IT750, showed less IOP reduction. These findings suggested that in non-glaucoma eyes without limited outflow facility, the relationship between anatomical metrics and the degree of IOP reduction is more straightforward than in eyes with glaucoma. Eyes with a preexisting small anterior chamber appeared to benefit more from anterior chamber deepening following lens removal. Prior studies have shown that eyes with a small anterior chamber, as measured by ACD, experience greater IOP reduction following cataract surgery [33,34]. ACarea, provides a more comprehensive representation of anterior chamber dimensions by incorporating both depth and width. Smaller ACarea has also been linked to greater postoperative IOP reduction [34]. In addition, Yang et al. demonstrated that the magnitude of IOP reduction was related to the increase in anterior chamber dimensions from pre- to post-operation [34]. Eyes with a smaller anterior segment tend to gain more chamber space after lens removal, resulting in a larger IOP reduction. Moreover, eyes with a thick peripheral iris might have a limited capacity for angle widening, resulting in less IOP reduction. A study by Lee et al. investigated the relationship between peripheral iris thickness and anterior chamber widening. Their results, in consistent with ours, showed that thin IT750 – rather than IT200 or maximum iris thickness –was associated greater anterior chamber widening after laser peripheral iridotomy [35]. These findings support the contribution of the peripheral iris contribute to angle crowding. The final model for the non-glaucoma group was clinically coherent, as it incorporated the most important clinical parameter (preIOP), a measure of central chamber (ACarea), and a peripheral angle-related parameter (IT750).

Of note, although CCT was not significantly associated with IOP change in univariable analysis, it was unexpectedly retained by LASSO selection most of the time. Its inclusion in the final multivariable models also consistently improved predictive performance. This suggests that CCT may offer additional explanatory value when considered in combination with other variables.

Our results support the recommendation that IOP reduction prediction models should be stratified by glaucoma status, as the relevant predictors and model performance differed markedly between the subgroups. The prediction of IOP reduction in glaucomatous eyes may depend less exclusively on anterior chamber dimensions. The stratification may also enhance their practical use in clinical practice. In addition, although 3D parameters may offer a more comprehensive view of anterior segment anatomy, the added complexity did not translate into superior predictive performance over 2D measurements in our dataset. Obtaining 3D parameters also involves greater technical difficulties to obtain sufficient image quality in all meridional scans. Therefore, a single high-resolution 2D imaging of horizontal meridian may be sufficient and reasonable for clinical use in terms of IOP prediction. Additional research is needed to determine other clinical roles of 3D parameters – for instance, distinguishing between different morphologic subtypes of angle closure.

This study has several strengths. First, it incorporates novel 3D parameters derived from AS-OCT, providing a more comprehensive assessment of anatomical predictors for IOP changes following cataract surgery. Second, the prospective design with controlled glaucoma medications—maintaining the same number of medications before surgery and during the one-month postoperative period—helps minimize medication-related confounding effects. Furthermore, all cataract surgeries were performed by a single surgeon, reducing variability associated with different surgical techniques.

The study also has some limitations. First, the prediction models for glaucoma and non-glaucoma subgroups were developed from a relatively small sample size, potentially affecting the robustness and generalizability of the results. The short follow-up duration of one month is another limitation; however, this was intentionally chosen because we generally maintain the same glaucoma medications during this early postoperative period, minimizing medication-induced IOP fluctuations. Prior studies also indicate that maximum IOP reduction typically occurs within the first week and remains relatively stable after 1 month, supporting the chosen timeframe [36–38]. However, some reports suggest that stabilization may occur later [39]. Therefore, further studies are needed to evaluate the prediction of long-term IOP control. Moreover, the dataset comprised solely Asian participants, who typically exhibit smaller anterior chambers and thicker irises, potentially limiting the generalizability of the findings to other ethnic groups. Lastly, the prediction models mainly focus on anatomical factors. Incorporating additional aspects such as biological function of trabecular outflow, ocular biomechanics and surgical variables (e.g., ultrasound power during phacoemulsification) might further improve predictive accuracy, particularly in the glaucoma subgroup. Lastly, although the internal validation utilizing bootstrap optimism correction was performed, external validation remains necessary to fully assess the predictive performance across broader populations.

In conclusion, both 3D and conventional 2D AS-OCT parameters demonstrated improved predictive value for IOP reduction following phacoemulsification compared to the preoperative IOP model. The predictive performance was particularly notable in the non-glaucoma subgroup. Nevertheless, the 2D and 3D models performed comparably in predicting IOP reduction.

## Supporting information

S1 Fig Distribution by angle and glaucoma status with diagnosis subtypes. (TIFF)

S1 Table Univariable analysis of the entire cohort. (PDF)

S2 Table Univariable analysis of the glaucoma subgroup. (PDF)

S3 Table Univariable analysis of the non-glaucoma subgroup. (PDF)

S4 Table Final predictive models. (PDF)
