# Supplementary figures and images for: Comparable performance of 3D and 2D anterior segment optical coherence tomography in predicting intraocular pressure reduction following cataract surgery
Source: PLoS One. 2026 Mar 25;21(3):e0345582. doi: 10.1371/journal.pone.0345582 (PMC13016306; doi:10.1371/journal.pone.0345582)

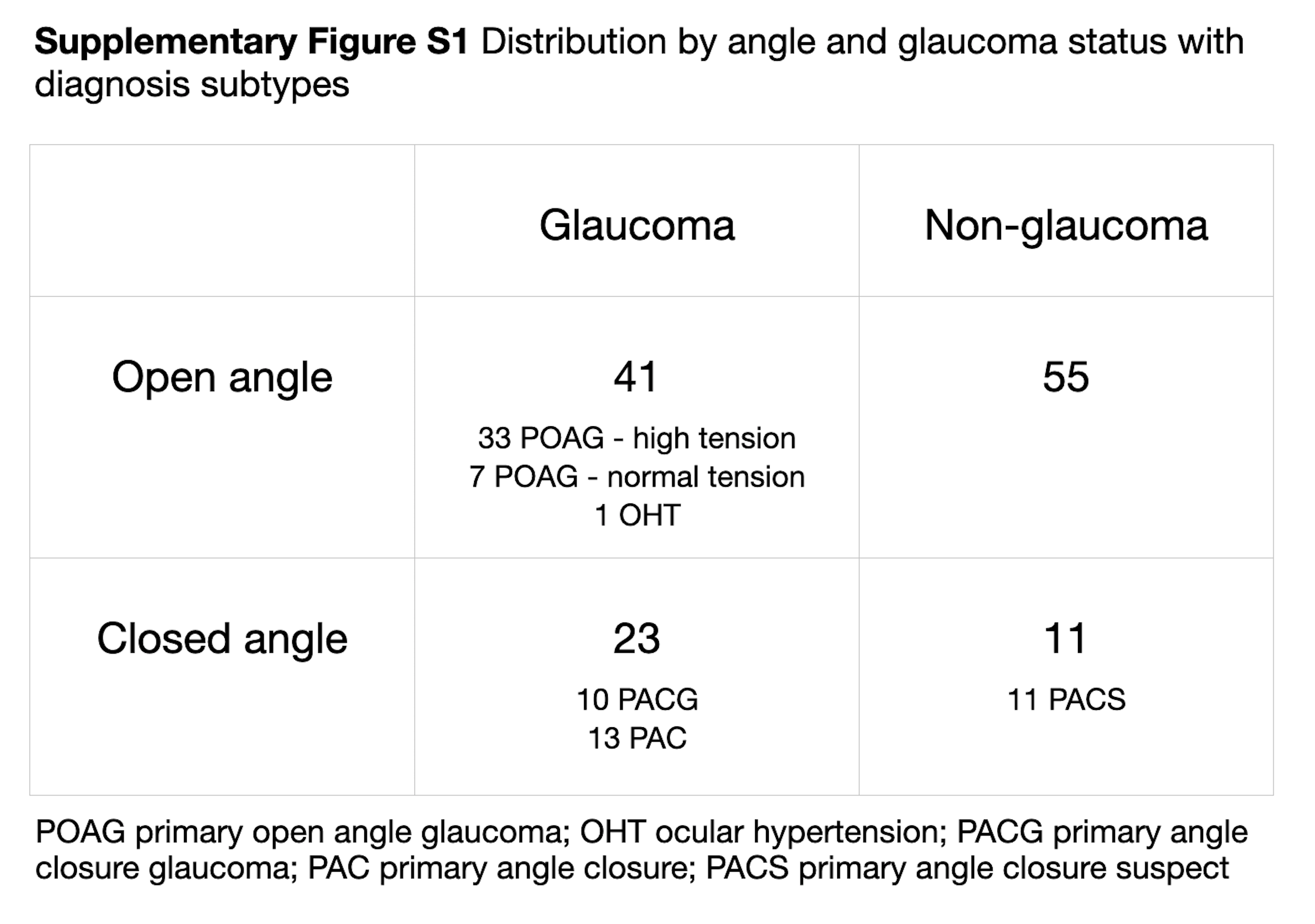

Supplement: S1 Fig — (TIFF) [file pone.0345582.s001.tiff]
