# Supplementary material for: Comparable performance of 3D and 2D anterior segment optical coherence tomography in predicting intraocular pressure reduction following cataract surgery
Source: PLoS One. 2026 Mar 25;21(3):e0345582. doi: 10.1371/journal.pone.0345582 (PMC13016306; doi:10.1371/journal.pone.0345582)
Supplement: S1 Table — (PDF) [file pone.0345582.s002.pdf]

Supplementary Table S2: Univariable analysis of the entire cohort

Variable	Mean / Count	SD / %	β coeff.	95% confidence interval		P-value
				Lower	Upper	
Clinical data						
Age, years	71.0	8.7	-0.075	-0.132	-0.017	**0.012**
Sex* (male)	42	40.8	-0.574	-1.777	0.628	0.346
preIOP, mmHg	14.192	3.671	-0.441	-0.655	-0.227	**<0.001**
Axial measurement						
ACD, mm	2.981	0.603	-0.140	-1.462	1.182	0.834
LT* (thick lens)	42	32.308	-0.502	-1.789	0.785	0.441
AL, mm	23.738	1.262	0.010	-0.556	0.577	0.971
CCT, μm	523.900	32.117	0.000	-0.018	0.017	0.965
LV, mm	0.452	0.489	0.346	-1.338	2.029	0.685
3D anterior segment morphometrics						
ACW-avg, mm	11.367	0.412	0.831	-0.701	2.362	0.285
ACarea-avg, mm^2	18.089	4.499	0.018	-0.174	0.210	0.854
ACarea-est†, mm^3	117.638	34.303	0.001	-0.025	0.026	0.959
AOD250-avg* (open)	78	60.0	0.135	-1.043	1.312	0.821
AOD500-avg* (open)	99	76.2	-0.612	-2.123	0.900	0.425
AOD750-avg* (open)	96	73.8	-1.078	-2.551	0.396	**0.150**
ARA250-avg* (open)	97	74.6	0.209	-1.206	1.624	0.771
ARA500-avg* (open)	95	73.1	-0.480	-1.897	0.938	0.504
ARA750-avg* (open)	95	73.1	-0.362	-1.807	1.082	0.621
TISA250-avg* (open)	75	57.7	0.396	-0.763	1.556	0.500
TISA500-avg* (open)	81	62.3	-0.039	-1.239	1.162	0.949
TISA750-avg* (open)	95	73.1	-0.402	-1.847	1.044	0.584
AOD250-est* (open)	100	76.9	-0.183	-1.769	1.403	0.819
AOD500-est* (open)	97	74.6	-0.278	-1.696	1.139	0.698
AOD750-est* (open)	94	72.3	-0.902	-2.311	0.508	0.208
ARA250-est* (open)	99	76.2	0.278	-1.197	1.753	0.710
ARA500-est* (open)	86	66.2	-0.039	-1.296	1.218	0.951
ARA750-est* (open)	87	66.9	-0.067	-1.342	1.208	0.917
TISA250-est* (open)	72	55.4	0.466	-0.680	1.611	0.423
TISA500-est* (open)	82	63.1	0.067	-1.143	1.277	0.913
TISA750-est* (open)	91	70.0	-0.297	-1.640	1.046	0.663
IT750-avg, mm	0.373	0.059	5.076	-3.745	13.897	0.257
IT2000-avg, mm	0.411	0.059	1.018	-8.729	10.764	0.837
IT750-est, mm^2	11.172	2.213	0.025	-0.300	0.350	0.881
IT2000-est, mm^2	9.371	1.596	0.034	-0.337	0.404	0.857
Iarea-avg, mm^2	1.518	0.217	-2.011	-4.992	0.970	**0.184**
Iarea-est‡, mm^3	35.189	4.314	0.002	-0.146	0.150	0.975

Icurv-avg, mm^2	0.175	0.095	0.205	-6.932	7.343	0.955
2D anterior segment morphometrics						
ACW-hoz, mm	11.305	0.428	1.082	-0.490	2.654	**0.176**
ACarea-hoz, mm^2	18.285	4.473	0.019	-0.172	0.209	0.847
AOD250-hoz* (open)	74	56.9	0.388	-0.767	1.543	0.507
AOD500-hoz* (open)	93	71.5	-0.441	-1.800	0.917	0.521
AOD750-hoz* (open)	95	73.1	-0.832	-2.269	0.606	0.254
ARA250-hoz* (open)	62	47.7	0.428	-0.715	1.571	0.460
ARA500-hoz* (open)	64	49.2	0.409	-0.734	1.552	0.480
ARA750-hoz* (open)	31	23.8	-0.490	-1.984	1.005	0.518
TISA250-hoz* (open)	63	48.5	0.341	-0.803	1.486	0.556
TISA500-hoz* (open)	78	60.0	0.263	-0.919	1.445	0.661
TISA750-hoz* (open)	75	57.7	-0.265	-1.449	0.919	0.658
IT750-hoz, mm	0.369	0.069	2.272	-5.929	10.472	0.585
IT2000-hoz, mm	0.401	0.067	0.847	-7.586	9.281	0.843
Iarea-hoz, mm^2	1.447	0.217	-1.249	-3.978	1.481	0.367
Icurv-hoz, mm	0.179	0.101	0.173	-6.214	6.560	0.957

Bold indicates p-value < 0.2; 3D = three-dimensional; 2D = two-dimensional; IOP = intraocular pressure; ACD = anterior chamber depth; LT = lens thickness; AL = axial length; CCT = central corneal thickness, LV = lens vault; ACW = anterior chamber width; ACarea = anterior chamber area; AOD = angle opening distance; ARA = angle recess area; TISA = trabecular iris space area; IT = iris thickness; Iarea = iris area; Icurv = iris curvature; -avg = average of 360-degree angle values; -est = estimation of circumferential area (for IT and AOD) or circumferential volume (for ACarea, Iarea, ARA, and TISA); -hoz = horizontal meridian (average of nasal and temporal sides); * binary factors - the value in parentheses indicates the represented category; † equivalent to anterior chamber volume; ‡ equivalent to iris volume
