# Supplementary material for: Comparable performance of 3D and 2D anterior segment optical coherence tomography in predicting intraocular pressure reduction following cataract surgery
Source: PLoS One. 2026 Mar 25;21(3):e0345582. doi: 10.1371/journal.pone.0345582 (PMC13016306; doi:10.1371/journal.pone.0345582)
Supplement: S2 Table — (PDF) [file pone.0345582.s003.pdf]

Supplementary Table S3: Univariable analysis of the glaucoma subgroup

Variable	Mean / Count	SD / %	β coeff.	95% confidence interval		P-value
				Lower	Upper	
Clinical data						
Age, years	70.6	8.2	-0.076	-0.175	0.023	**0.129**
Sex* (male)	24	47.1	-0.714	-2.341	0.914	0.384
preIOP, mmHg	14.438	4.375	-0.297	-0.452	-0.141	**<0.001**
Axial measurement						
ACD, mm	2.817	0.647	-0.608	-2.233	1.018	0.458
LT* (thick lens)	23.000	35.938	0.784	-1.117	2.685	0.413
AL, mm	23.390	0.945	-0.698	-1.353	-0.044	**0.037**
CCT, μm	521.063	31.895	0.000	-0.027	0.027	0.990
LV, mm	0.625	0.515	1.482	-0.412	3.376	**0.123**
3D anterior segment morphometrics						
ACW-avg, mm	11.369	0.401	1.238	-0.801	3.277	0.229
ACarea-avg, mm^2	16.756	4.531	-0.106	-0.292	0.081	0.261
ACarea-est†, mm^3	108.868	34.309	-0.014	-0.038	0.010	0.252
AOD250-avg* (open)	32	50.0	-0.406	-2.065	1.253	0.626
AOD500-avg* (open)	44	68.8	-1.205	-3.272	0.863	0.249
AOD750-avg* (open)	41	64.1	-2.005	-3.932	-0.078	**0.042**
ARA250-avg* (open)	43	67.2	-0.063	-2.101	1.975	0.951
ARA500-avg* (open)	41	64.1	-0.716	-2.669	1.238	0.467
ARA750-avg* (open)	42	65.6	-0.708	-2.745	1.330	0.490
TISA250-avg* (open)	30	46.9	0.116	-1.523	1.754	0.888
TISA500-avg* (open)	33	51.6	-0.449	-2.124	1.226	0.594
TISA750-avg* (open)	41	64.1	-0.784	-2.754	1.186	0.430
AOD250-est* (open)	44	68.8	-0.477	-2.625	1.671	0.658
AOD500-est* (open)	43	67.2	-0.488	-2.449	1.472	0.620
AOD750-est* (open)	40	62.5	-1.592	-3.470	0.287	**0.095**
ARA250-est* (open)	43	67.2	-0.063	-2.101	1.975	0.951
ARA500-est* (open)	36	56.3	-0.583	-2.327	1.160	0.506
ARA750-est* (open)	37	57.8	-0.632	-2.410	1.147	0.480
TISA250-est* (open)	28	43.8	0.266	-1.365	1.896	0.746
TISA500-est* (open)	33	51.6	-0.386	-2.063	1.290	0.647
TISA750-est* (open)	39	60.9	-0.670	-2.534	1.194	0.475
IT750-avg, mm	0.371	0.052	-0.379	-17.102	16.344	0.964
IT2000-avg, mm	0.416	0.057	1.561	-14.342	17.463	0.845
IT750-est, mm^2	11.009	2.160	-0.247	-0.799	0.306	0.376
IT2000-est, mm^2	9.492	1.553	0.018	-0.524	0.559	0.949
Iarea-avg, mm^2	1.501	0.205	-1.947	-6.357	2.463	0.381
Iarea-est‡, mm^3	35.088	3.927	0.025	-0.176	0.227	0.801

Icurv-avg, mm^2	0.165	0.082	7.423	-1.589	16.435	**0.105**
2D anterior segment morphometrics						
ACW-hoz, mm	11.312	0.414	1.663	-0.695	4.020	**0.164**
ACarea-hoz, mm^2	16.947	4.505	-0.106	-0.291	0.079	0.257
AOD250-hoz* (open)	30	46.9	0.053	-1.585	1.691	0.949
AOD500-hoz* (open)	38	59.4	-0.941	-2.720	0.837	0.294
AOD750-hoz* (open)	41	64.1	-1.802	-3.714	0.111	**0.064**
ARA250-hoz* (open)	23	35.9	0.444	-1.114	2.003	0.571
ARA500-hoz* (open)	24	37.5	-0.008	-1.578	1.561	0.992
ARA750-hoz* (open)	10	15.6	-1.270	-2.686	0.145	**0.078**
TISA250-hoz* (open)	27	42.2	0.311	-1.293	1.916	0.699
TISA500-hoz* (open)	33	51.6	-0.011	-1.693	1.672	0.990
TISA750-hoz* (open)	28	43.8	-1.258	-2.787	0.271	**0.105**
IT750-hoz, mm	0.367	0.063	-0.796	-14.515	12.923	0.908
IT2000-hoz, mm	0.405	0.065	3.157	-9.575	15.888	0.622
Iarea-hoz, mm^2	1.435	0.205	-0.578	-4.375	3.219	0.762
Icurv-hoz, mm	0.168	0.090	6.560	-2.482	15.602	**0.152**

Bold indicates p-value < 0.2; 3D = three-dimensional; 2D = two-dimensional; IOP = intraocular pressure; ACD = anterior chamber depth; LT = lens thickness; AL = axial length; CCT = central corneal thickness, LV = lens vault; ACW = anterior chamber width; ACarea = anterior chamber area; AOD = angle opening distance; ARA = angle recess area; TISA = trabecular iris space area; IT = iris thickness; Iarea = iris area; Icurv = iris curvature; -avg = average of 360-degree angle values; -est = estimation of circumferential area (for IT and AOD) or circumferential volume (for ACarea, Iarea, ARA, and TISA); -hoz = horizontal meridian (average of nasal and temporal sides); * binary factors - the value in parentheses indicates the represented category; † equivalent to anterior chamber volume; ‡ equivalent to iris volume
