# Supplementary material for: Comparable performance of 3D and 2D anterior segment optical coherence tomography in predicting intraocular pressure reduction following cataract surgery
Source: PLoS One. 2026 Mar 25;21(3):e0345582. doi: 10.1371/journal.pone.0345582 (PMC13016306; doi:10.1371/journal.pone.0345582)
Supplement: S3 Table — (PDF) [file pone.0345582.s004.pdf]

Supplementary Table S4: Univariable analysis of the non-glaucoma subgroup

Variable	Mean / Count	SD / %	β coeff.	95% confidence interval		P-value
				Lower	Upper	
Clinical data						
Age, years	70.5	9.7	-0.075	-0.147	-0.002	**0.044**
Sex* (male)	18	34.6	-0.372	-2.336	1.593	0.707
preIOP, mmHg	13.955	2.842	-0.772	-1.009	-0.535	**<0.001**
Axial measurement						
ACD, mm	3.140	0.515	0.403	-1.933	2.738	0.732
LT* (thick lens)	19.000	28.788	-1.868	-3.500	-0.236	**0.026**
AL, mm	24.075	1.435	0.270	-0.500	1.041	0.486
CCT, μm	526.652	32.335	-0.001	-0.026	0.023	0.920
LV, mm	0.285	0.399	-1.106	-4.187	1.975	0.476
3D anterior segment morphometrics						
ACW-avg, mm	11.366	0.425	0.481	-1.805	2.767	0.676
ACarea-avg, mm^2	19.382	4.102	0.149	-0.209	0.507	0.408
ACarea-est†, mm^3	126.143	32.331	0.015	-0.033	0.063	0.542
AOD250-avg* (open)	46	69.7	0.659	-0.944	2.262	0.415
AOD500-avg* (open)	55	83.3	0.109	-2.022	2.240	0.919
AOD750-avg* (open)	55	83.3	0.109	-2.022	2.240	0.919
ARA250-avg* (open)	54	81.8	0.500	-1.193	2.193	0.557
ARA500-avg* (open)	54	81.8	-0.315	-2.210	1.581	0.741
ARA750-avg* (open)	53	80.3	-0.017	-1.907	1.873	0.985
TISA250-avg* (open)	45	68.2	0.635	-0.941	2.210	0.424
TISA500-avg* (open)	48	72.7	0.333	-1.309	1.976	0.687
TISA750-avg* (open)	54	81.8	-0.009	-2.012	1.993	0.993
AOD250-est* (open)	56	84.8	0.129	-2.102	2.359	0.909
AOD500-est* (open)	54	81.8	-0.111	-2.134	1.912	0.913
AOD750-est* (open)	54	81.8	-0.111	-2.134	1.912	0.913
ARA250-est* (open)	56	84.8	0.718	-1.125	2.560	0.439
ARA500-est* (open)	50	75.8	0.540	-1.184	2.264	0.534
ARA750-est* (open)	50	75.8	0.540	-1.184	2.264	0.534
TISA250-est* (open)	44	66.7	0.614	-0.941	2.168	0.433
TISA500-est* (open)	49	74.2	0.511	-1.157	2.180	0.543
TISA750-est* (open)	52	78.8	0.066	-1.737	1.869	0.942
IT750-avg, mm	0.375	0.066	8.225	-2.308	18.757	**0.124**
IT2000-avg, mm	0.407	0.060	0.837	-11.823	13.497	0.895
IT750-est, mm^2	11.331	2.269	0.256	-0.046	0.557	**0.095**
IT2000-est, mm^2	9.255	1.640	0.059	-0.480	0.598	0.828
Iarea-avg, mm^2	1.534	0.228	-2.160	-6.209	1.889	0.290
Iarea-est‡, mm^3	35.286	4.686	-0.015	-0.225	0.196	0.891

Icurv-avg, mm^2	0.184	0.106	-4.218	-13.193	4.757	0.351
2D anterior segment morphometrics						
ACW-hoz, mm	11.297	0.444	0.602	-1.579	2.784	0.583
ACarea-hoz, mm^2	19.581	4.070	0.152	-0.199	0.502	0.391
AOD250-hoz* (open)	44	66.7	0.682	-0.872	2.235	0.384
AOD500-hoz* (open)	55	83.3	0.109	-2.022	2.240	0.919
AOD750-hoz* (open)	54	81.8	0.398	-1.644	2.440	0.698
ARA250-hoz* (open)	39	59.1	0.348	-1.227	1.922	0.661
ARA500-hoz* (open)	40	60.6	0.738	-0.804	2.280	0.342
ARA750-hoz* (open)	21	31.8	-0.146	-2.252	1.960	0.890
TISA250-hoz* (open)	36	54.5	0.322	-1.268	1.912	0.687
TISA500-hoz* (open)	45	68.2	0.495	-1.068	2.058	0.529
TISA750-hoz* (open)	47	71.2	0.685	-0.917	2.288	0.396
IT750-hoz, mm	0.371	0.075	4.267	-6.378	14.912	0.426
IT2000-hoz, mm	0.398	0.070	-0.924	-12.374	10.525	0.872
Iarea-hoz, mm^2	1.459	0.229	-1.833	-5.650	1.985	0.341
Icurv-hoz, mm	0.189	0.110	-4.193	-11.521	3.136	0.257

Bold indicates p-value < 0.2; 3D = three-dimensional; 2D = two-dimensional; IOP = intraocular pressure; ACD = anterior chamber depth; LT = lens thickness; AL = axial length; CCT = central corneal thickness, LV = lens vault; ACW = anterior chamber width; ACarea = anterior chamber area; AOD = angle opening distance; ARA = angle recess area; TISA = trabecular iris space area; IT = iris thickness; Iarea = iris area; Icurv = iris curvature; -avg = average of 360-degree angle values; -est = estimation of circumferential area (for IT and AOD) or circumferential volume (for ACarea, Iarea, ARA, and TISA); -hoz = horizontal meridian (average of nasal and temporal sides); * binary factors - the value in parentheses indicates the represented category; † equivalent to anterior chamber volume; ‡ equivalent to iris volume
