# Supplementary material for: Comparable performance of 3D and 2D anterior segment optical coherence tomography in predicting intraocular pressure reduction following cataract surgery
Source: PLoS One. 2026 Mar 25;21(3):e0345582. doi: 10.1371/journal.pone.0345582 (PMC13016306; doi:10.1371/journal.pone.0345582)
Supplement: S4 Table — (PDF) [file pone.0345582.s005.pdf]

Supplementary Table S5: Final predictive models

Cohorts	3D models				2D models			
	Predictors	Coeff.	Std err.	p-value	Predictors	Coeff.	Std err.	p-value
**All subjects**	preIOP	-0.484	0.067	<0.001	preIOP	-0.480	0.067	<0.001
	age	-0.068	0.029	0.023	age	-0.079	0.029	0.006
	sex (male)	-1.194	0.547	0.031	sex (male)	-1.183	0.547	0.032
	AOD750-avg (open)	-1.350	0.554	0.016	AOD750-hoz (open)	-1.261	0.546	0.023
	IT750-avg	4.868	4.194	0.248				
	CCT	0.014	0.008	0.077	CCT	0.013	0.008	0.096
	Iarea-avg	-1.738	1.168	0.139				
	ACW-avg	1.051	0.654	0.110	ACW-hoz	1.041	0.609	0.090
	Constant	-6.613	9.500	0.488	Constant	-5.940	8.894	0.505
**Glaucoma**	preIOP	0.334	0.086	<0.001	preIOP	-0.335	0.085	<0.001
	age	-0.059	0.045	0.189	age	-0.049	0.045	0.279
	AOD750-avg (open)	-1.892	0.799	0.021	AOD750-hoz (open)	-1.706	0.775	0.032
	AL	-0.760	0.483	0.121	AL	-0.875	0.451	0.057
	ACW-avg	1.919	1.093	0.084	ACW-hoz	2.397	1.001	0.020
	CCT	0.015	0.012	0.196	CCT	0.017	0.011	0.151
	Constant	-3.121	13.669	0.820	Constant	-7.322	13.449	0.588
**Non-glaucoma**	preIOP	-0.819	0.105	<0.001	preIOP	-0.829	0.104	<0.001
	ACarea-avg	0.161	0.077	0.041	ACarea-hoz	0.188	0.074	0.014
	IT750-avg	5.877	4.663	0.212	IT750-hoz	5.666	3.955	0.157
	CCT	0.017	0.010	0.089	CCT	0.018	0.010	0.074
	Constant	-3.860	5.431	0.480	Constant	-4.521	5.461	0.411
